# The Effect of BC Sealer, AH-Plus and Dorifill on Push-out Bond Strength of Fiber Post 

**DOI:** 10.22037/iej.v12i4.15863

**Published:** 2017

**Authors:** Fatemeh Dibaji, Elahe Mohammadi, Farzaneh Farid, Fatemeh Mohammadian, Pegah Sarraf, Mohammad Javad Kharrazifard

**Affiliations:** a *Department of Endodontics, Dental School, Tehran University of Medical Sciences, International Campus, Tehran, Iran; *; b *Dental Student, Dental School, Tehran University of Medical Sciences, International Campus, Tehran, Iran; *; c * Department of Prosthodontics, Dental School, Tehran University of Medical Sciences, International Campus , Tehran, Iran; *; d *Dental Research Center, Tehran University of Medical Sciences, Tehran, Iran*

**Keywords:** Bond Strength, Endodontic Sealer, Fiber Post, Resin Cement

## Abstract

**Introduction:**

Dentinal canal walls are in direct contact with endodontic sealers prior to post space preparation and luting cements after post space preparation. This direct contact may affect the bond strength of intraradicular posts to root dentin. This study aimed to assess the effect of three different sealers on the bond strength of fiber posts to root dentin.

**Methods and Materials:**

The canals of 56 extracted single-rooted human premolars after selection and decoronation were prepared. For obturation of the canals, specimens were randomly divided into four groups (*n*=14) according to the type of sealer used in conjunction with gutta-percha: group 1 (control) without any sealer; group 2 with AH-Plus sealer (resin based); group 3 with Dorifill sealer (ZOE-based); and group 4 with BC Sealer (calcium silicate-based). Nine mm-deep post space was prepared in the canal of each specimen. Intraradicular fiber posts were cemented using dual-cure resin cement (Panavia F2.0). Sections of 1 mm thickness were made at the coronal, middle and apical thirds of the post space of each specimen. The push-out bond strength of post to root dentin was measured in a universal testing machine. The data were analyzed using one-way ANOVA and post-hoc Tukey’s test.

**Results:**

The mean push-out bond strength in the coronal third was significantly lower in Dorifill group compared to AH-Plus (*P*=0.004). This value was significantly lower in BC Sealer group than AH-Plus (*P*=0.000) and control group (*P*=0.03). In middle and apical thirds, the mean push-out bond strength was not significantly different among the four groups (*P*=0.407, *P*=0.065, respectively). The mean push-out bond strength was significantly lower in apical than coronal third in AH-Plus group (*P*=0.001).

**Conclusion:**

Application of BC Sealer and Dorifill decreased the mean push-out bond strength of intracanal post to root dentin in the coronal third in comparison to AH-Plus.

## Introduction

Generally, gutta-percha in conjunction with a sealer is routinely used as filling materials for root canal treatment [1, 2]. Sealers facilitate root canal obturation due to their flowability, seal the lateral and apical accessory canals [3] and provide an optimal adaptation to root dentin [4]. Among the sealers used in root canal treatment, bioceramic sealers such as BC sealer are becoming increasingly popular due to their ability to bond to dentin surface and formation of hydroxyapatite. Bioceramic sealers are premixed, injectable, radiopaque and hydrophilic and have an alkaline pH. Moreover, these sealers use the moisture of dentinal tubules to initiate and accomplish their setting reactions [5, 6].

After completion of root canal treatment, proper restoration of tooth is required to restore its function and esthetics [7]. Most endodontically treated teeth have lost a large portion of their structure due to caries, previous restorations or fracture and require an indirect restoration, which most of the time needs insertion of intraradicular posts [8, 9]. These posts play a significant role in retention and durability of final restoration. Of different types of available intracanal posts, fiber posts have been recommended as a suitable alternative to metal posts due to their flexibility being close to that of dentin. This characteristic decreases the risk of root fracture in endodontically treated teeth [7, 10]. 

Based on previous studies, success of endodontically treated teeth with intracanal posts depends on proper selection of filling material, sealer and type of intracanal post [7, 11]. Many of the fiber post failures occur between the root canal wall and adhesive resin cement [12, 13]. Moreover, it has been shown that type of sealer can affect the bond strength of fiber posts to root dentin [14-17].

A previous study has shown that zinc oxide-eugenol-based sealers decrease the retention of fiber post [18]. The drawbacks of these sealers include weakening the chemical bonding between the root canal dentin wall and also inhibition of composite polymerization due to the eugenol content [19]. Since BC Sealer has been recently introduced to the market and studies on the effect of this sealer on bond strength of fiber posts to root dentin are lacking, this study sought to assess the effect of BC Sealer (calcium silicate-based sealers), AH-Plus (resin based) and Dorifill (ZOE-based) on push-out bond strength of fiber post to root dentin. The null hypothesis was the kind of sealer would have no effect on the push out bond strength of fiber posts cemented with dual cured resin cement.

## Materials and Methods

A total of 56 single-rooted and single canal human premolar teeth, extracted due to periodontal problems, were collected for this study. Radiographs were taken to ensure the presence of a single canal. For disinfection, the teeth were immersed in 0.5% chloramine-T solution for one week. External surfaces of teeth were cleaned from debris and necrotic tissue using an ultrasonic scaler (Piezo201, Kavo Dental Excellence, Biberach, Germany). The teeth were evaluated under a light microscope (SMX800, Nikon Co., NY, USA) under 10× magnification to exclude teeth with cracks. Tooth crowns were cut at the cementoenamel junction using a diamond disc and low speed handpiece under water coolant so that 13±1 mm of root length remained. Working length was determined by subtracting 1 mm from the length of a #10 K-file (Mani Inc., Tochigi, Japan) after observing its tip at the apical foramen. 

Flaring the coronal third of the root canal was performed using #2 to 4 Gates Glidden drills (Mani Inc., Tochigi, Japan). All root canals were prepared up to #45 K-file. Root canals were irrigated using 2.5% NaOCl solution after using each file. After root canal preparation, smear layer was removed using 1 mL of 17% ethylenediaminetetraacetic acid (EDTA) (Cina Bartar, Tehran, Iran) for one min, 3 mL of 5.25% NaOCl and a final rinse with 5 mL of distilled water.

The root canals were then dried with paper points (Ariadent, Asia Chemi Teb Co, Tehran, Iran). According to the sealer used for filling of the root canal system, the roots were randomly divided into four groups of 14 as follows: Group 1 (Control), gutta-percha without sealer; group 2, gutta-percha and AH-Plus sealer (DeTrey/Dentsply, Konstanz, Germany); group 3, gutta-percha and Dorifill sealer (Dorident Company, Austria) and group 4, Gutta-percha and BC Sealer (EndoSequence, Maillefer, Savannah, USA). Root canal sealers were prepared as recommended by the manufacturers and root canals were filled using cold lateral compaction technique. Then the orifice of the root canals were sealed with temporary filling material (Cavit, 3M ESPE, Germany). 

The teeth were incubated in 100% humidity at 37^°^C for one week. Then, 9 mm depth post space was prepared using a #2 peeso reamer (Mani Inc., Tochigi, Japan) and a #2 drill (Innopost Premier Anatomic, Innotech, Italy) in each root canal. The canals were then rinsed with copious water and dried with paper points. Fiber posts were cemented into the root with Panavia F2.0 resin cement (Kuraray, Medical, Japan) according to the manufacturer’s instructions. Fiber posts were placed deep into the canal using finger pressure and resin cement was polymerized for 20 sec using VALO light curing unit (Ultradent Product Inc., South Jordan, USA). The roots were then incubated in 100% humidity at 37^°^C for 24 h. In order to cut cross-sectional slices, the roots were mounted in blocks containing clear polyester resin. After polymerization, dentin discs were sectioned with 1 mm thickness at the coronal, middle and apical thirds of the created post space using a Mecatome with diamond disc (Mecatome T 201 A; PERSI, France) under copious water irrigation. The push-out bond strength was measured in a universal testing machine (Zwick/Roell, Zo50, Ulm, Germany) with a crosshead speed of 0.5 mm/min (from the apical towards the coronal). Using the following formula push-out bond strength was calculated: Maximum load (N)/area of fiber post (mm^2^). The area of fiber post was calculated using *π*(*R*+*r*)[(*h*^2^+(*R*-*r*)^2^]/2 where *R*(mm), *r*(mm) and *h*(mm) are larger radius, smaller radius and the thickness of the root section, respectively. The push-out bond strength data were converted from Newtons to Megapascals (MPa). Mode of bond failure was determined under a stereomicroscope (KyKy, Maillefer, China) under 10× magnification. The percentage of each mode of failure in each group was calculated. 

Data were analyzed using one-way ANOVA and post-hoc Tukey’s test. The mode of failure was classified into four types: 1) adhesive between the post and resin cement, 2) mixed with resin cement covering 0-50% of the post diameter, 3) mixed with resin cement covering 50-100% of post diameter and 4) cohesive in dentin.

## Results

Table 1 presents the minimum, maximum, mean push-out bond strength and standard deviation (SD) of the groups. The one-way ANOVA showed that the effect of type of sealer on the mean push-out bond strength was significant. Thus, the mean bond strength in four groups was separately evaluated and compared in coronal, middle and apical thirds of the prepared post space. Since the difference in the mean push-out bond strength was significant among the groups (*P*=0.001), in the coronal third, pairwise comparisons were carried out and revealed that the mean push-out bond strength of fiber post to root dentin was significantly lower where Dorifill was used compared to AH-Plus (*P*=0.004). Also, the mean push-out bond strength of fiber post to root dentin in BC Sealer group was significantly lower than that of AH-Plus group (*P*=0.000) and that in control group (*P*=0.03). In the middle and apical third region, the mean push-out bond strength of post to root dentin was not significantly different among the four groups (*P*=0.407) and (*P*=0.065), respectively. 

In AH-Plus group, the mean push-out bond strength in apical was significantly lower than coronal third (*P*=0.001). However, the mean push-out bond strength was not significantly different in the coronal, middle or apical thirds in the Dorifill (*P*=0.321), BC Sealer (*P*=0.358) and control (*P*=0.321) groups.

Table 2 presents the results of the predominating type of failure in each group. The prevalence of mixed fractures and adhesive cement-dentine failure was verified in all of the groups.

## Discussion

The present study evaluated the effect of three different endodontic sealers on push-out bond strength of fiber post to root dentin. The result showed that BC sealer and Dorifill significantly had lower push out bond strength in coronal third in comparison with AH-Plus and control groups. So the null hypothesis was rejected. 

**Table 1:** Mean (SD) of push-out bond strength of various sealers at coronal, middle and apical thirds of root canal in MPa

**Site**	**Sealers**	**Min**	**Max**	**Mean (SD)**
**Coronal**	Control	2.00	12.01	5.33 (3.42)
	BC sealer	0.69	6.74	2.60 (1.81)
	AH-Plus	1.83	12.70	6.98 (3.14)
	Dorifill	1.26	8.19	3.42 (1.59)
**Middle**	Control	1.19	10.00	4.15 (2.90)
	BC sealer	1.18	9.25	3.54 (2.80)
	AH-Plus	1.14	10.02	5.11 (2.39)
	Dorifill	1.30	9.61	3.96 (2.43)
**Apical**	Control	0.74	7.74	3.74 (2.16)
	BC sealer	0.22	8.29	2.37 (2.01)
	AH-Plus	0.67	5.78	3.22 (1.59)
	Dorifill	1.31	8.64	4.37 (2.50)

**Table 2:** Types of failures in each group

	**Adhesive (Cement-Dentin)**	**Mix (0-50)%**	**Mix (50-100)%**	**Cohesive**
**No sealer (Control)**	50.01% )21(	45.23% )19)	4.76% (2)	-
**Resin-based sealer (AH-Plus)**	40.47% )17(	7.14% )3(	52.38% (22 )	-
**Calcium silicate-based sealer (BC sealer)**	61.90% )26(	28.57% )12(	2.38% (1)	-
**Eugenol-based sealer (Dorifill)**	66.66% )28(	19.04% )8(	16.66% (7)	-

Endodontically treated teeth are commonly restored with fiber post and resin luting cement [20]. Fiber posts are cemented into the post space created in the root canal system. Resin-reinforced fiber posts are suitable alternatives to conventional posts [21, 22]. Resin cements can form a mono-block with root canal walls [22]. Retention is provided by the contact between root dentin, luting cement and intracanal post [22]. Thus, the success of a fiber post depends on proper bond of post to root dentin [22, 23]. Cementation of fiber posts with resin cements yields optimal results in terms of high retention, low microleakage and high resistance of root to fracture [23].

On the other hand, type of the endodontic sealer can affect the bond between resin cement and root dentin, so as the eugenol inside the zinc oxide eugenol sealer may modify the resin cement and decrease the bond strength of resin cement to root dentin. So the sealer can affect the strength of fiber post to root dentin [14, 17].To measure the bond strength of materials there are various techniques such a conventional tensile test, pull-out and the push-out tests. The advantage of the latter seems to be more close simulation of the clinical condition [24].

Based on the results of the present study, the mean push-out bond strength in the coronal third was significantly lower where Dorifill was used in comparison to AH-Plus, which was in agreement with the results of previous studies [16, 25-27]. Moreover, the mean push-out bond strength in the coronal third in BC Sealer group was lower than that of AH-Plus. However, Reyhani *et al.* [26] showed that MTA-Fillapex (calcium silicate-based sealer) had no significant difference with AH-Plus (resin based) in this regard.

In the middle third region, the push-out bond strength was not significantly different among the three sealer groups of the present study, which was in agreement with the findings of Reyhani *et al.* [26] and Gundogar *et al.* [16], using Ever Stick post with Duo-link resin cement. However, it was in contrast to Gundogar *et al. *results [16] that had used DT Light and Transluma posts with Duo-link resin cement.

In the apical third of the root, no significant difference was noted in bond strength among the three sealers used, which was in accordance with the results of Gundogar *et al.* [16], when Ever Stick post with Duo-link resin cement was used and in contrast to it when Transluma post with Duo-link resin cement was employed.

The present study showed that type of sealer affected the push-out bond strength of fiber post to root dentin in the coronal third of the post space. In the coronal third of the root, tubular density, diameter of dentinal tubules and the created post space are greater than those in the apical third. Moreover, access of etchant, adhesive and curing light is greater in coronal areas compared to the apical third of the root [28]. Furthermore, contamination of dentinal walls with sealer and gutta-percha after root filling is greater in ideally prepared post spaces compared to larger post spaces as shown on scanning electron microscopic (SEM) micrographs. Despite the adverse effects of endodontic sealers on retention of fiber post to root dentin, extending the post space improves the bond strength of self-adhesive resin cements used as luting agents to dentin [29]. In the current study, ideal post spaces were prepared; thus, there is a possibility that after post space preparation, sealer remnants on root dentin of the coronal third had a greater impact on bond strength compared to that in the middle and apical thirds. It may be assumed that due to better elimination of sealers from the root dentin in the middle and apical thirds, the bond strength was found to be the same in these areas in the four groups. 

It has been reported that the retentive strength of fiber posts to root dentin increases by increasing in diameter of posts (creating a larger post space) when ZOE-based sealers are used. This is probably due to the removal of a thicker layer of dentin affected by sealers and increased surface area for resin cement bond [30]. Because infiltration of eugenol content of ZOE-based sealers into dentinal tubules affects the setting of resin cements and decreases their bond strength due to the properties of phenolic compounds in eugenol [17, 31]. 

After post space preparation, sealers or eugenol remaining on root canal dentinal walls must be eliminated in order not to decrease or prevent the polymerization reactions of luting cements [32]. Cohen *et al.* [27] showed that epoxy resin does not interfere with free radicals initiating composite resin polymerization. Thus, resin-based sealers do not adversely affect the bond of resin cements. The remnants of AH-26 resin sealer on dentinal walls of the created post space in the root canal can improve the bond of resin cement [32].

Cecchin *et al.* [25] stated that high bond strength between resin based sealer to resin based cement may be due to the affinity of epoxy resin sealer components to this cement components. Several studies have shown that during root canal retreatment, complete removal of BC Sealer from the root canal system is difficult [33-35]. Moreover, studies have shown differences in bond strength of AH-Plus and BC sealers to root dentin. Some studies have shown that the bond strength of BC sealer to root dentin was higher than that of AH-Plus [36, 37], whilst others stated BC sealer and AH-Plus had similar bond strength to root dentin [38] or BC sealer had a lower bond strength to root dentin than AH-Plus [39, 40]. Differences in bond strength may be due to differences in study designs and methodologies (method of filling, sealer brand and its composition) [41].

In present study in AH-Plus sealer group the mean push-out bond strength was significantly lower in the apical thirds than coronal third, in agreement to the results of Gundogar *et al.* [16], with DT Light post and Duo-link resin cement and in contrast to the results of Cecchin *et al.* [25].

This study has shown that in Dorifill group, the mean push-out bond strength in the coronal, middle and apical thirds was not significantly different, which was in agreement with the results of previous studies [16, 25]. In our study control group did not have a significant difference with other groups in the middle and apical thirds of post in terms of push-out bond strength, which was similar to the results of Cecchin *et al. *[25]; but had a significant difference with BC Sealer group at the coronal third, which was in line with the result of studies on MTA Fillapex and contradicted with the result of studies on iRoot sealers [26, 42].

Considering the fact that no previous study has assessed the bond strength of fiber post to root dentin with the use of BC Sealer, the current results were compared with those on MTA-Fillapex [26, 43, 44] and iRoot [42, 45] for root filling. However, none of the afore-mentioned studies divided the post space into coronal, middle and apical thirds for further assessment and comparison of bond strength in these regions. Furthermore, the difference between the results of our study and those of other studies can be due to the differences in the materials used such as type of cement and fiber post used. Removal of smear layer was considered in this study. Because it has been shown that smear layer plays an important role in evaluating the bond strength of materials to root canal dentin and influences the adhesion of the self-etching luting system such as Panavia [46]. Also, presence of a thick smear layer in post space can decrease the bond strength of resin cement [28]. In general, creating a post space free of any contamination is among the most important factors in achieving a strong bond when resin cements are applied [47]. Attempts must be made to eliminate sealer residues from the post space to enhance the bond strength of fiber post and resin cement to dentinal walls. 

## Conclusion

Within the limitation of this study, application of BC Sealer and Dorifill decreased the mean bond strength of fiber post to root dentin compared to AH-Plus sealer in the coronal third. This effect was not seen in the middle and apical thirds.

## References

[1] Apicella MJ, Loushine RJ, West LA, Runyan DA (1999). A comparison of root fracture resistance using two root canal sealers. Int Endod J.

[2] Rajput JS, Jain RL, Pathak A (2004). An evaluation of sealing ability of endodontic materials as root canal sealers. J Indian Soc Pedod Prev Dent.

[3] Hargreaves KM, Cohen S, Berman LH (2011). Cohen's pathways of the pulp.

[4] Sabadin N, Böttcher DE, Hoppe CB, Santos RBd, Grecca FS (2014). Resin-based sealer penetration into dentinal tubules after the use of 2% chlorhexidine gel and 17% EDTA: in vitro study. Braz J Oral Sci.

[5] Koch K, Brave D (2009). Bioceramic technology-the game changer in endodontics. Endodontic Practice US.

[6] Zhang H, Shen Y, Ruse ND, Haapasalo M (2009). Antibacterial activity of endodontic sealers by modified direct contact test against Enterococcus faecalis. J Endod.

[7] Teixeira CdS, Pasternak‐Junior B, Borges AH, Paulino SM, Sousa‐Neto MD (2008). Influence of endodontic sealers on the bond strength of carbon fiber posts. J Biomed Mater Res B Appl Biomater.

[8] Boone KJ, Murchison DF, Schjndler WG, Walker WA (2001). Post retention: the effect of sequence of post-space preparation, cementation time, and different sealers. J Endod.

[9] Cagidiaco MC, Goracci C, Garcia-Godoy F, Ferrari M (2008). Clinical studies of fiber posts: a literature review. Int Endod J.

[10] Bateman G, Ricketts D, Saunders W (2003). Fibre-based post systems: a review. Br Dent J.

[11] Schwartz RS, Murchison DF, Walker WA (1998). Effects of eugenol and noneugenol endodontic sealer cements on post retention. J Endod.

[12] Gomes MF, Botta SB, Matos AB, Netto NG (2012). The interference of the cleaning procedure of root walls with two different solvents on the adhesion of fiberglass intraradicular posts. J Contemp Dent Pract.

[13] Serafino C, Gallina G, Cumbo E, Ferrari M (2004). Surface debris of canal walls after post space preparation in endodontically treated teeth: a scanning electron microscopic study. Oral Surg Oral Med Oral Pathol Oral Radiol Endod.

[14] Cobankara F, Unlu N, Cetin A, Ozkan H (2008). The effect of different restoration techniques on the fracture resistance of endodontically-treated molars. Oper Dent.

[15] Ghanadan K, Ashnagar S, Omrani LR, Mirzaee M (2015). Effect of different endodontic sealers on push-out bond strength of fiber posts. Braz J Oral Sci.

[16] Gündoğar M, Gençoğlu N (2012). The effect of different root canal sealers on the bond strength of different fiber post systems.

[17] Aleisa K, Alghabban R, Alwazzan K, Morgano SM (2012). Effect of three endodontic sealers on the bond strength of prefabricated fiber posts luted with three resin cements. J Prosthet Dent.

[18] Altmann AS, Leitune VC, Collares FM (2015). Influence of Eugenol-based Sealers on Push-out Bond Strength of Fiber Post Luted with Resin Cement: Systematic Review and Meta-analysis. J Endod.

[19] Aggarwal V, Singla M, Miglani S, Kohli S (2012). Effect of different root canal obturating materials on push-out bond strength of a fiber dowel. J Prosthodont.

[20] Pashley DH, Tay FR, Breschi L, Tjäderhane L, Carvalho RM, Carrilho M, Tezvergil-Mutluay A (2011). State of the art etch-and-rinse adhesives. Dent Mater.

[21] Schwartz RS, Robbins JW (2004). Post placement and restoration of endodontically treated teeth: a literature review. J Endod.

[22] Qualtrough A, Mannocci F (2003). Tooth-colored post systems: a review. Oper Dent.

[23] Demiryürek EÖ, Külünk Ş, Yüksel G, Saraç D, Bulucu B (2010). Effects of three canal sealers on bond strength of a fiber post. J Endod.

[24] Goracci C, Tavares AU, Fabianelli A, Monticelli F, Raffaelli O, Cardoso PC, Tay F, Ferrari M (2004). The adhesion between fiber posts and root canal walls: comparison between microtensile and push-out bond strength measurements. Eur J Oral Sci.

[25] Cecchin D, Farina A, Souza M, Carlini‐Júnior B, Ferraz C (2011). Effect of root canal sealers on bond strength of fibreglass posts cemented with self‐adhesive resin cements. Int Endod J.

[26] Reyhani MF, Ghasemi N, Rahimi S, Milani AS, Omrani E (2016). Effect of Different Endodontic Sealers on the Push-out Bond Strength of Fiber Posts. Iran Endod J.

[27] Cohen BI, Volovich Y, Musikant BL, Deutsch AS (2002). The effects of eugenol and epoxy-resin on the strength of a hybrid composite resin. J Endod.

[28] Topcu FT, Erdemir U, Sahinkesen G, Mumcu E, Yildiz E, Uslan I (2012). Push-out bond strengths of two fiber post types bonded with different dentin bonding agents. J Biomed Mater Res B Appl Biomater.

[29] Othman HI, Elshinawy MI, Abdelaziz KM (2013). Retention of fiber posts to the optimally and over-prepared dowel spaces. J Adv Prosthodont.

[30] Izadi A, Azarsina M, Kasraei S (2013). Effect of eugenol-containing sealer and post diameter on the retention of fiber reinforced composite posts. J Conserv Dent.

[31] Dias LLL, Giovani AR, Sousa S, Corrêa YT, Vansan LP, Alfredo E, Sousa-Neto MD, Paulino SM (2009). Effect of eugenol-based endodontic sealer on the adhesion of intraradicular posts cemented after different periods. J Appl Oral Sci.

[32] Mosharraf R, Zare S (2014). Effect of the type of endodontic sealer on the bond strength between fiber post and root wall dentin. J Dent (Tehran).

[33] Hess D, Solomon E, Spears R, He J (2011). Retreatability of a bioceramic root canal sealing material. J Endod.

[34] Ersev H, Yılmaz B, Dinçol M, Dağlaroğlu R (2012). The efficacy of ProTaper Universal rotary retreatment instrumentation to remove single gutta‐percha cones cemented with several endodontic sealers. Int Endod J.

[35] Ma J, Al-Ashaw AJ, Shen Y, Gao Y, Yang Y, Zhang C, Haapasalo M (2012). Efficacy of ProTaper Universal Rotary Retreatment System for Gutta-percha Removal from Oval Root Canals: A Micro–Computed Tomography Study. J Endod.

[36] DeLong C, He J, Woodmansey KF (2015). The effect of obturation technique on the push-out bond strength of calcium silicate sealers. J Endod.

[37] Pawar A, Pawar S, Kfir A, Pawar M, Kokate S (2015). Push‐out bond strength of root fillings made with C‐Point and BC sealer versus gutta‐percha and AH Plus after the instrumentation of oval canals with the Self‐Adjusting File versus WaveOne. Int Endod J.

[38] Shokouhinejad N, Gorjestani H, Nasseh AA, Hoseini A, Mohammadi M, Shamshiri AR (2013). Push‐out bond strength of gutta‐percha with a new bioceramic sealer in the presence or absence of smear layer. Aust Endod J.

[39] Gade VJ, Belsare LD, Patil S, Bhede R, Gade JR (2015). Evaluation of push-out bond strength of endosequence BC sealer with lateral condensation and thermoplasticized technique: An in vitro study. J Conserv Dent.

[40] Ozkocak I, Sonat B (2015). Evaluation of effects on the adhesion of various root canal sealers after Er: YAG laser and irrigants are used on the dentin surface. J Endod.

[41] Wang Z (2015). Bioceramic materials in endodontics. Endod topics.

[42] Sukuroglu E, Aslan Y, Nagas E, Canay S, Senyilmaz DP (2015). Effect of root canal sealers on the push-out bond strengths of tooth-colored posts to root dentine. Acta Odontol Scand.

[43] Reyhani MF, Ghasemi N, Rahimi S, Milani AS, Mokhtari H, Shakouie S, Safarvand H (2014). Push-out bond strength of Dorifill, Epiphany and MTA-Fillapex sealers to root canal dentin with and without smear layer. Iran Endod J.

[44] Adl AR, Sobhnamayan F, Shojaee NS, Azizi S (2016). A Comparison of Push-Out Bond Strength of Two Endodontic Sealers to Root Canal Dentin: An in Vitro Study. J Clin Exp Dent.

[45] Ersahan S, Aydin C (2010). Dislocation resistance of iRoot SP, a calcium silicate–based sealer, from radicular dentine. J Endod.

[46] Gu XH, Mao CY, Liang C, Wang HM, Kern M (2009). Does endodontic post space irrigation affect smear layer removal and bonding effectiveness?. Eur J Oral Sci.

[47] Davis S, O'CONNELL B (2007). The effect of two root canal sealers on the retentive strength of glass fibre endodontic posts. J Oral Rehabil.

